# Intracellular soluble α‐synuclein oligomers reduce pyramidal cell excitability

**DOI:** 10.1113/JP271968

**Published:** 2016-03-24

**Authors:** Timothy J. Kaufmann, Paul M. Harrison, Magnus J. E. Richardson, Teresa J. T. Pinheiro, Mark J. Wall

**Affiliations:** ^1^ School of Life Sciences; ^2^ Warwick Systems Biology CentreUniversity of WarwickCoventryUK

## Abstract

**Key points:**

- The presynaptic protein α‐synuclein forms aggregates during Parkinson's disease.
- Accumulating evidence suggests that the small soluble oligomers of α‐synuclein are more toxic than the larger aggregates appearing later in the disease.
- The link between oligomer toxicity and structure still remains unclear.
- In the present study, we have produced two structurally‐defined oligomers that have a similar morphology but differ in secondary structure.
- These oligomers were introduced into neocortical pyramidal cells during whole‐cell recording and, using a combination of experimentation and modelling, electrophysiological parameters were extracted.
- Both oligomeric species had similar effects on neuronal properties reducing input resistance, time constant and increasing capacitance. The net effect was a marked reduction in neuronal excitability that could impact on network activity.

**Abstract:**

The presynaptic protein α‐synuclein (αSyn) aggregates during Parkinson's disease (PD) to form large proteinaceous amyloid plaques, the spread of which throughout the brain clinically defines the severity of the disease. During early stages of aggregation, αSyn forms soluble annular oligomers that show greater toxicity than much larger fibrils. These oligomers produce toxicity via a number of possible mechanisms, including the production of pore‐forming complexes that permeabilize membranes. In the present study, two well‐defined species of soluble αSyn oligomers were produced by different protocols: by polymerization of monomer and by sonication of fibrils. The two oligomeric species produced were morphologically similar, with both having an annular structure and consisting of approximately the same number of monomer subunits, although they differed in their secondary structure. Oligomeric and monomeric αSyn were injected directly into the soma of pyramidal neurons in mouse neocortical brain slices during whole‐cell patch clamp recording. Using a combined experimental and modelling approach, neuronal parameters were extracted to measure, for the first time in the neocortex, specific changes in neuronal electrophysiology. Both species of oligomer had similar effects: (i) a significant reduction in input resistance and the membrane time constant and (ii) an increase in the current required to trigger an action potential with a resultant reduction in the firing rate. Differences in oligomer secondary structure appeared to produce only subtle differences in the activity of the oligomers. Monomeric αSyn had no effect on neuronal parameters, even at high concentrations. The oligomer‐induced fall in neuronal excitability has the potential to impact both network activity and cognitive processing.

## Key points

- The presynaptic protein α‐synuclein forms aggregates during Parkinson's disease.
- Accumulating evidence suggests that the small soluble oligomers of α‐synuclein are more toxic than the larger aggregates appearing later in the disease.
- The link between oligomer toxicity and structure still remains unclear.
- In the present study, we have produced two structurally‐defined oligomers that have a similar morphology but differ in secondary structure.
- These oligomers were introduced into neocortical pyramidal cells during whole‐cell recording and, using a combination of experimentation and modelling, electrophysiological parameters were extracted.
- Both oligomeric species had similar effects on neuronal properties reducing input resistance, time constant and increasing capacitance. The net effect was a marked reduction in neuronal excitability that could impact on network activity.

**Abbreviations**
αSyn:  α‐synuclein
AUC: analytical ultracentrifugation
CD: circular dichroism
DAPI: 4′,6‐diamidino‐2‐phenylindole
EIF: exponential integrate‐and‐fire
PBS: phosphate‐buffered saline
PD: Parkinson's disease
TBS: Tris‐buffered saline
TBST: Tris‐buffered saline + Tween20
TEM: transmission electron microscopy
TTL5: thick‐tufted layer 5

## Introduction

α‐Synuclein (αSyn), a 14 kDa protein found abundantly throughout the brain (Vivacqua *et al*. 2011), was one of the first proteins to be pathologically associated with Parkinson's disease (PD). This association came initially from identifying αSyn in the Lewy bodies of PD patients. Indeed, the detection of these αSyn‐containing plaques, in various brain regions, remains a clinical hallmark for classifying the progression of several neurodegenerative disorders that are collectively termed synucleinopathies. In some cases of familial PD, point mutations in αSyn (A53T, A30P, E46K, H50Q and G51D) (Polymeropoulos *et al*. 1997; Kruger *et al*. 1998; Zarranz *et al*. 2004; Lesage *et al*. 2013; Proukakis *et al*. 2013) and gene multiplications (Fuchs *et al*. 2007) promote the early onset of symptoms as a result of an increase in the propensity of αSyn to aggregate. However, increasing evidence suggests that soluble, ring‐like oligomers are more neurotoxic and better correlate with PD pathology than the larger insoluble aggregates into which they form (Haass & Selkoe, 2007; Winner *et al*. 2011).

Native αSyn exists predominantly in the cytosol as either an unfolded monomer (Fauvet *et al*. 2012) or as a helically folded tetramer (Bartels *et al*. 2011; Wang *et al*. 2011 *a*). There have been many studies investigating the structure and activity of αSyn oligomers, with oligomerization protocols varying considerably with respect to the protein concentration (Volles *et al*. 2001; Danzer *et al*. 2007), incubation conditions (Lashuel *et al*. 2002; Lorenzen *et al*. 2014), buffer content (Hoyer *et al*. 2002) and the use of inducers such as metal ions (Binolfi *et al*. 2006; Brown, 2009) or dopamine (Cappai *et al*. 2005). Unsurprisingly, the oligomers produced with these different conditions show variability in terms of their size, structure and toxicity. Clearly, data on the biological activity of the oligomers become difficult to interpret if the structure is not defined.

Both *in vitro* and *in vivo* studies describe the ability of αSyn oligomers to form membrane inserting pore‐complexes that can induce cell death through various mechanisms, including membrane permeabilization (Volles & Lansbury, 2002; Tosatto *et al*. 2012), Ca^2+^ influx (Danzer *et al*. 2007), synaptic alterations (Diogenes *et al*. 2012; Pacheco *et al*. 2015) and mitochondrial dysfunction (Guardia‐Laguarta *et al*. 2014). Step changes in membrane conductance have been observed after incubating αSyn with either planar bilayers (Kim *et al*. 2009; Schmidt *et al*. 2012; Tosatto *et al*. 2012) or cell cultures (Feng *et al*. 2010; Mironov, 2015). Many of these investigations, however, attribute the observed electrophysiological changes to oligomers that have not been structurally characterized.

In addition to motor dysfunction, clinically diagnosed PD patients exhibit a wide range of debilitating non‐motor symptoms that have a devastating impact on quality of life (Chaudhuri *et al*. 2006). More than 80% of Parkinson's patients develop PD dementia, which can include sleep disorders arising from αSyn aggregation in the basal forebrain (Boeve, 2013), as well as neuropsychiatric symptoms, including depression, dementia, hallucinations and emotional disturbances (Chaudhuri *et al*. 2006), as a result of deposition in the deep layers of the neocortex (Magen *et al*. 2012; Müller & Bohnen, 2013). However, compared to other areas involved in cognitive function, little research has been carried out investigating the impact of αSyn oligomers on neocortical neurons.

In the present study, we provide the structural characterization of two soluble oligomeric species of αSyn and then inject these oligomers directly into thick‐tufted layer 5 (TTL5) pyramidal neurons in neocortical slices. We decided to study this cell type, rather than SNC dopaminergic neurons, because it is affected in late stage PD (Braak *et al*. 2003) and we can employ an efficient method for neuronal model identification (Badel *et al*. 2008). Subsequently, we can produce an in‐depth analysis of toxicity‐induced changes in neuronal parameters giving insight into the mechanism of oligomer activity. Over time, neurons injected with these two oligomeric species displayed a similar reduction in input resistance and membrane time constant compared to either control or monomer‐injected neurons. Consequentially, the amount of current needed for spike initiation was elevated significantly. As expected, the firing rate was also reduced, although this effect took longer to develop, suggesting that it is not just a consequence of the fall in input resistance. These effects on neuron excitability are important because they could potentially change network dynamics.

## Methods

### Expression and purification of recombinant αSyn^E46K,Y39W^

The pGS‐21a vector (GenScript Corporation, Piscataway, NJ, USA) containing recombinant human αSyn with two point mutations, E46K and Y39W, was transformed into BL21* Rosetta *Escherichia coli* cells by heat shock and then expression was induced with 1 mm isopropyl d‐thiogalactopyranoside. Cells were harvested after 4 h of expression and resuspended in cold lysis buffer (10 mm Tris, 1 mm EDTA, 1 mm phenylmethylsulphonyl fluoride, pH 8.0). Cells were lysed by probe sonication (3 × 30 s at 60 % power) and centrifuged (10,000 *g* for 15 min at 4°C) to remove debris. The supernatant was boiled for 10 min and re‐centrifuged (20,000 *g* for 20 min at 4°C). Proteins, including αSyn, were removed from the heat treated supernatant by ammonium sulphate precipitation (50% saturation). The precipitated protein was resuspended in Tris buffer (10 mm Tris/HCl, pH 8.0) and re‐solubilized overnight by dialysis using SnakeSkin Dialysis Tubing (Thermo Scientific, Waltham, MA, USA). The αSyn‐containing sample was loaded onto a 10 ml Source 30Q anion exchange column (GE Healthcare, Little Chalfont, UK) equilibrated with Tris buffer at a flow rate of 2 ml min^−1^. Proteins were eluted with a linear NaCl gradient (0–700 mm; 2 ml min^−1^). The fractions containing αSyn were collected between 280–340 mm NaCl, concentrated by lyophilization and loaded onto a HiPrep 26/60 Sephacryl S‐300 High Resolution gel filtration column (Amersham Biosciences, Piscataway, NJ, USA). Fractions containing αSyn were determined by SDS‐PAGE and western blotting. Purified αSyn was dialysed into 10 mm sodium phosphate buffer (pH 7.4), aliquoted into 200 μl volumes, flash‐frozen in liquid nitrogen and stored at −20°C until use.

### Oligomerization of αSyn

Oligomeric species of αSyn were generated by two different methods. The first method was modified from a protocol described previously (Lorenzen *et al*. 2014). In brief, lyophilized samples of monomeric αSyn were suspended in 300–400 μl of phosphate‐buffered saline (PBS) (in mm: 137 NaCl, 2.7 KCl, 8.1 Na_2_HPO_4_ and 1.5 KH_2_PO_4_; pH 7.4) + 0.01% NaN_3_ to give a final concentration of between 10–12 mg ml^−1^. Samples were incubated at 37°C with shaking (900 rpm) for 3 h in an Eppendorf Thermomixer Compact (Fisher Scientific Co., Pittsburgh, PA, USA). Insoluble aggregates were removed by ultracentrifugation at 100,000 *g* (10 min at 4°C) and the supernatant (containing soluble oligomer and monomer) was loaded onto a Superdex 200 gel filtration column (GE Healthcare) equilibrated with PBS, at a flow rate of 0.5 ml min^−1^. The oligomer‐containing fractions were collected and concentrated using Amicon Ultra 0.5 ml centrifugal filters (molecular weight cut‐off 3 kDa) (Sigma‐Aldrich, St Louis, MO, USA). These oligomers, which were produced directly from monomeric αSyn, are termed *mOligomers*.

The second method produced oligomers by fragmentation of large fibrillar aggregates. Fibrilization was carried out under the same conditions as described for *mOligomer*s with the incubation time extended overnight. Insoluble fibrils were washed thoroughly to remove any remaining monomer and *mOligomer*. Fibrils were resuspended in 300–400 μl of PBS + 0.01 % azide and sonicated for 20 s at 60% power. The sonicated sample was ultracentrifuged, purified and concentrated as described above. These oligomers, which were recovered from fibril fragmentation, are termed *fOligomers*.

Both types of oligomer were stored at 4°C and used within 24 h for structural and electrophysiological investigations.

### Dot blot analysis

Blotted aliquots of αSyn (10 × 2 μl) were dried on nitrocellulose before being blocked for 1 h in Tris‐buffered saline (TBS: 10 mm Tris; 150 mm NaCl; pH 7.5) + Tween20 (TBST) containing 2% dried skimmed milk (Marvel; Premier Foods, St Albans, UK). Primary antibodies were diluted 1:1000 and secondary antibodies were diluted 1:5000 in the blocking buffer. Mouse anti‐αSyn antibody (catalogue number 610786; BD Biosciences, Clontech, Palo Alto, CA, USA) was used to detect the presence of αSyn regardless of structure, the secondary for which was anti‐mouse IgG conjugated to alkaline phosphatase (Apase; Promega, Madison, WI, USA). Rabbit polyclonal A11 antibody (catalogue number AHB0052; Invitrogen, Carlsbad, CA, USA) was used to specifically detect oligomeric αSyn (Kayed *et al*. 2003), the secondary for which was anti‐rabbit IgG conjugated to APase (Promega). Before and after the addition of secondary antibodies, the paper was washed thoroughly in TBST to remove non‐specific binding. A final wash in TBS was carried out to remove Tween20 prior to the addition of Western Blue (Promega), a substrate for APase, that enabled visualization of the target protein.

### Circular dichroism (CD) and fluorescence spectroscopy

Far‐UV CD spectra (190–260 nm) were measured with a J‐815 spectropolarimeter (Jasco, Chelmsford, UK) using 1 mm path length quartz cuvettes, a scanning speed of 100 nm min^−1^, a time constant of 1 s and bandwidth of 1 nm. Spectra were collected at a resolution of 0.5 nm and an accumulation of 16–32 scans were averaged per spectrum. CD spectra were analysed using DichroWeb (Whitmore & Wallace, 2004, 2008), comparing spectra with a reference data set (Set 7) using the SELCON3 analysis algorithm (Sreerama & Woody, 1993).

Fluorescence spectra were recorded with a spectrofluorometer (Photon Technology International, Stanmore, UK) using a 4 mm path length quartz cuvette. The sample was excited at 295 nm (2 nm bandwidth) and the emission spectra were recorded from 300 to 450 nm (2 nm bandwidth) with a resolution of 1 nm and an integration time of 1 s. Each spectrum was the average of four scans.

### Analytical ultracentrifugation (AUC)

Protein samples were prepared <24 h in advance and analysed using a ProteomeLab XL‐I (Beckman Coulter, Fullerton, CA, USA) protein characterization system. Samples were diluted in PBS to give a final concentration of 0.1–0.2 mg ml^−1^ as determined by a single scan (*A*_280_) at 3000 rpm. The sedimentation coefficient was determined from a total of 285 scans per sample. AUC was carried out at 20 ± 0.1°C in sedimentation velocity mode. The heterogeneity of oligomer samples was assessed by comparing the peak width at half‐height.

### Transmission electron microscopy (TEM)

Copper electronmicroscopy grids, 400 mesh with formvar carbon support film (Agar Scientific, Stansted, UK), were hydrophilized in a K100x Glow Discharger (Quorum Technologies, Lewes, UK). Protein samples were adsorbed over 60 s, stained using 2% uranyl acetate and then washed with distilled water. Images were collected on a JEM2011 CyroTransmission electron microscope (Jeol Inc., Peabody, MA, USA) with 200 kV acceleration, and analysed semi‐automatically using ImageJ software (NIH, Bethesda, MD, USA). To define oligomer structure, measurements were made from at least three preparations of each oligomer species, with multiple electronmicroscopy images captured from each preparation. Over 20 TEM images were analysed for each oligomer with ∼50 particles analysed per image.

### Preparation of neocortical slices

All experiments were approved by the Animal Welfare and Ethical Review Body (AWERB) at the University of Warwick. In accordance with the UK Animals (Scientific Procedures) Act (1986), male B6CBF1 mice (postnatal days, P28–35) were killed by cervical dislocation and decapitated. The brain was rapidly removed and cut down the midline. Parasagittal neocortical brain slices (300 μm) were cut at an angle of +15° such that the blade cut from the surface of the neocortex towards the caudal border of the neocortex (ensuring the integrity of layer V pyramidal cell dendrites) using a Microm HM 650 V microslicer (Thermo Scientific). Slices were prepared using an ice‐cold cutting solution (in mm: 127 NaCl, 1.9 KCl, 8 MgCl_2_, 0.5 CaCl_2_, 1.2 KH_2_PO_4_, 26 NaHCO_3_ and 1 d‐glucose). Slices were transferred to a Gibbs chamber containing standard artificial cerebrospinal fluid (in mm: 1 MgCl_2_ and 2 CaCl_2_) bubbled with 95% O_2_/5% CO_2_, and incubated at 34°C for 60 min and then at room temperature (20–22°C) until use.

### Whole‐cell patch clamp recordings from pyramidal cells

A slice was transferred to the recording chamber, submerged in artificial cerebrospinal fluid and perfused at a constant flow rate of 3 ml min^−1^ (32°C). Slices were visualized using an BX51W1 microscope (Olympus, Tokyo, Japan) with IR‐DIC optics and a KP‐M1A CCD camera (Hitachi, Tokyo, Japan). Single or paired whole‐cell patch clamp recordings were made from pyramidal neurons using patch pipettes (4–8 MΩ resistance) manufactured from thick walled borosilicate glass (Harvard Apparatus, Edenbridge, UK) containing (in mm): 135 potassium gluconate, 7 NaCl, 10 Hepes, 0.5 EGTA, 2 ATP, 0.3 GTP, 10 phosphocreatine and 0.05 Alexa Fluor 488 hydrazide, 300 mosm (pH 7.2). Various αSyn species were added to the intracellular recording solution prior to patch clamp recording to give a final concentration of 0.5 μm (5 % v/v). In control cells, the vehicle PBS (5 % v/v) was added to the intracellular solution.

The voltage responses from current clamped neurons were recorded using a Multiclamp 700B amplifier (Molecular Devices, Sunnyvale, CA, USA) and digitized at 20 kHz using a Digidata 1440a (Molecular Devices). Data was acquired using pCLAMP software (Clampex, version 10; Molecular Devices). The liquid junction potential (calculated as ∼13 mV) was uncompensated. TTL5 pyramidal cells were identified based on their location in the layered neocortex, somata size and dendritic extent. During the recording, neurons were labelled with the fluorescent dye Alexa Fluor 488 to enable confirmation of the cell type and to ensure an intact apical dendrite.

### Immunostaining and confocal imaging

After patch clamp recording, brain slices were immediately fixed in 4 % paraformaldehyde for at least 1 h at 4°C, after which the slice was washed repeatedly with PBS. Slices were incubated for 1 h at room temperature in blocking buffer (1 % BSA, 0.4 % Triton X‐100 in PBS) before being suspended in either anti‐αSyn or A11 antibodies (for details, see section on Dot blot analysis), 1:100 dilution in blocking buffer, for 1 h at room temperature followed by overnight incubation at 4°C. The primary antibodies were aspirated and the slices washed thoroughly in PBS (5 × 10 min) prior to the addition of secondary antibodies (1:100 in blocking buffer): Alexa Fluor 568 goat anti‐mouse IgG (H+L) for anti‐αSyn staining or Alexa Fluor 647 chicken anti‐rabbit IgG (H+L) for A11 staining (#A11004 and #A21443, respectively; Molecular Probes, Carlsbad, CA, USA). Slices were incubated for 4 h at room temperature and washed in PBS afterwards. Slices were also stained with the nuclear stain 4′,6‐diamidino‐2‐phenylindole (DAPI) (1 μg ml^−1^ in blocking buffer). Slices were mounted on Polylysine adhesion slides (Thermo Scientific) and preserved in Vectashield medium (Vector Laboratories, Burlingame, CA, USA). Slices were stored at 4°C before imaging with confocal microscopy (TCS SP5; Leica Microsystems, Wetzlar, Germany).

### Fluorescence conjugation

For a subset of neurons, αSyn‐Dylight 594 conjugated monomer was added to the intracellular solution. A sample of αSyn monomer was tagged using a Dylight labelling kit (Thermo Scientific) in accordance with the mnufacturer's instructions. In brief, monomeric αSyn stock was diluted to 1 mg ml^−1^ in PBS + 0.05 m borate buffer and added to 65 μg of lyophilized Dylight 594 NHS ester. The mixture was protected from light and left to stand for 4 h at room temperature. Unbound ester was removed using the purification resin and microcentrifuge spin columns provided. Monomeric αSyn was found to be labelled at a ratio of 2.57 mol dye (mol protein)^–1^. Labelled αSyn was aliquoted, flash frozen and stored at −20°C until use.

### Stimulation protocols

To extract the electrophysiological properties of recorded neurons, both step and more naturalistic, fluctuating currents were injected at 8 min intervals (start‐to‐start) for a total duration of 32 min.

### Standard *I*–*V* protocol

The standard *I*–*V* relationship was obtained by the injection of step currents; starting between −600 to −400 pA, and incrementing by 100–200 pA until a regular firing pattern (6–12 Hz) was induced. A plot of step current against average voltage response around the resting potential was used to measure the input resistance (gradient of fitted line).

### Dynamic *I*–*V* protocol

The dynamic *I*–*V* curve was generated from the neuronal response to a continuously injected waveform (noisy current), which reflects ongoing synaptic activity. The dynamic *I*–*V* curve, defined by the average transmembrane current as a function of voltage, can be used to efficiently parameterize neurons and generate reduced neuron models that accurately mimic the cellular response. The complete method has been described previously (Badel *et al*. 2008); for the dynamic *I*–*V* computer code, see also Harrison *et al*. (2015). A brief account is given below.

Injected noisy current traces were constructed from the summed numerical output of two Ornstein–Uhlenbeck processes (Uhlenbeck & Ornstein, 1930) with time constants τ_fast_ = 3 ms and τ_slow_ = 10 ms. These stochastic waveforms mimic the background post‐synaptic activity resulting from activation of AMPA and GABA_A_ receptor channels. Two sets of variances were applied to the waveform (low; σ_fast_ = σ_slow_ = 0.18 and high; σ_fast_ = 0.36, σ_slow_ = 0.25), both with a DC bias of 0.06 (relative units), to give two different current traces (LowV‐06 and HighV‐06). Each trace had a duration of 40 s and was preceded and followed by 5 s of zero current (null stimulus). The traces were multiplied by a gain factor (300–800 pA) when injected to give a desired firing rate of 5–15 Hz. In a small number of experiments, the gain factor was kept constant at 400 pA throughout the time course to investigate the effects of αSyn on firing rate. For each time point, both the low and high variance traces were injected in series and then averaged to give the neuronal parameters.

The dynamic transmembrane current (*I*_ion_) can be calculated as:

(1) $$I_{\mathrm{ion}}\left(V,t\right)+I_{\mathrm{noise}}=I_{\mathrm{inj}}\left(t\right)-C\frac{dV}{dt}$$

for which the injected current (*I*_inj_) is known beforehand, the derivative (d*V*/d*t*) can be calculated from the experimentally measured voltage and the capacitance (*C*) is attained from a minimum variance procedure (Badel *et al*. 2008). A scatter plot of the transmembrane current against voltage illustrates the dynamic relationship between the two, with the effects of weak background synaptic activity and other sources of high‐frequency variability being accounted for as intrinsic noise (*I*_noise_) (Badel *et al*. 2008). Averaging the transmembrane current in 1 mV bins removes the time dependence of *I*_ion_ (*V*,*t*) to yield the typical ionic current and a particular voltage, and so defines the dynamic *I*–*V* curve (*I*_dyn_).

(2) $$I_{\mathrm{dyn}}\;\left(V\right)=\;Mean\left[I_{\mathrm{ion}}\left(V,t\right)\right]$$

Following earlier studies (Badel *et al*. 2008; Harrison *et al*. 2015), we found that the exponential integrate‐and‐fire model (Fourcaud‐Trocme *et al*. 2003) provided an excellent fit to the dynamic *I*–*V* curve. The exponential integrate‐and‐fire (EIF) model is characterized by a voltage forcing term *F*(*V*) that is related to the *I*_dyn_ as:

(3) $$F\;\left(V\right)=\frac{-I_{\mathrm{dyn}}\left(V\right)}{C}\;$$

where the steady‐state forcing function *F*(*V*) for the EIF model is given as:

(4) $$\begin{array}{c} F\;\left(V\right)=\frac{1}{\tau }\left(\;E-V+\Delta_{T}exp\;\left(\frac{V-V_{T}}{\Delta_{T}}\right)\right)=-\;\frac{I_{\mathrm{dyn}}\;\left(V\right)}{C} \end{array}$$

The EIF model has four parameters: membrane time constant (τ), resting potential (E), spike‐initiation threshold (V_T_) and spike‐onset sharpness (Δ_T_), which describes the voltage range over which an action potential initiates.

Pre‐spike parameters were calculated using a dynamic *I*–*V* curve fitted to the EIF model. Dynamic *I*–*V* curves were constructed solely from the pre‐spike voltage response (subthreshold and run up to spike) with all data falling within a 200 ms window after each spike being excluded from analysis.

### Percentage spike match

Parameters were fed into the EIF model to simulate a voltage response to the same noisy current inputs. The accuracy of these parameters was then tested by comparing the simulated response with the experimentally recorded voltage trace. The number of matching spikes, aligning to within ± 5 ms, was typically 60–90%.

### Statistical analysis

Using either the standard or dynamic *I*–*V* methods, parameters were extracted from neurons with different treatments of αSyn and over a range of time points. Individual treatments were compared across different time points using repeated measures ANOVA. At set time points, different αSyn treatments were analysed using two‐tailed *t* tests with Bonferoni correction for multiple comparisons. All neuronal parameters are presented as the mean with either the SD or SEM.

## Results

### Characterization of αSyn oligomers

**Figure 1:**
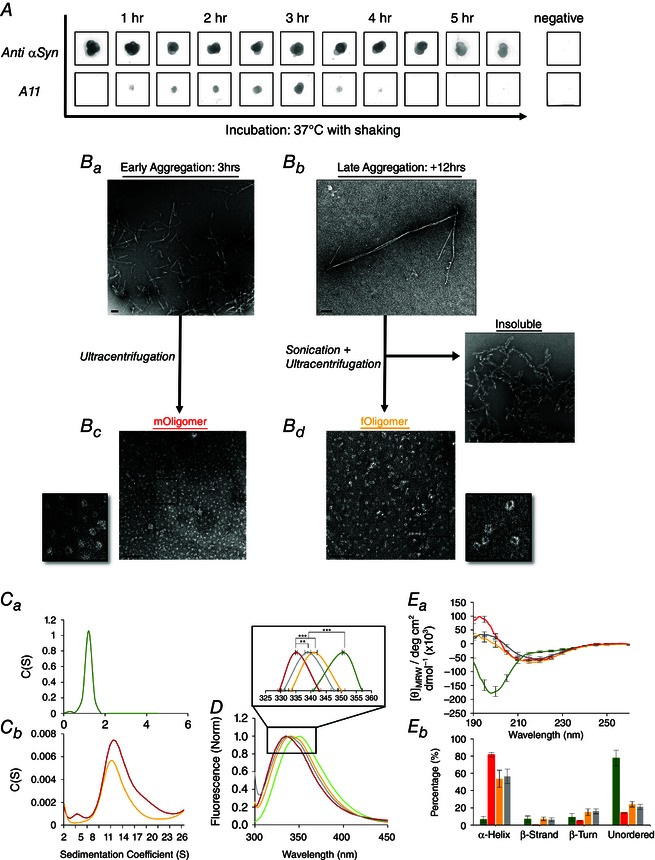
**Formation of αSyn oligomers and their structural characterization** *A*, dot blots probed with A11 antibody are used to monitor the aggregation of monomeric αSyn over time (negative control: BSA). *Ba*, TEM imaging at an A11‐positive time point (3 h) reveals a mixture of early‐stage aggregates comprising monomers, oligomers and protofibrils. *Bb*, if aggregation is allowed to continue for longer (over 4 h), A11 immunoreactivity is lost and large fibrils are formed. *Bc*, the supernatant from ultracentrifuged early‐stage aggregates provides a sample of soluble, ring‐like oligomers, termed *mOligomers*. *Bd*, sonication of late‐stage fibrils also recovers soluble, ring‐like oligomers (*fOligomers*) (TEM scale bars = 100 nm; insets = 25 nm). *Ca* and *Cb*, the size and sample heterogeneity of monomer and both oligomers was determined by ATC. *D*, the degree of blue shift exhibited by Trp39 in αSyn monomer, oligomers and fibrils, determined by fluorescence spectroscopy, provides information about the local structure. *Ea*, the secondary structure of αSyn populations was analysed with CD spectroscopy. *Eb*, the percentage structure was determined using DichroWeb. In all plots: αSyn monomers (green), *mOligomers* (red), *fOligomers* (yellow) and fibrils (grey).

For the production of αSyn oligomers, we used recombinant αSyn with two point mutations: E46K (a mutation found in familial PD that accelerates aggregation) (Choi *et al*. 2004) and Y39W to enable fluorescence spectroscopy. The latter was introduced because αSyn does not have any native tryptophan (Trp) residues and has been shown not to affect either αSyn secondary structure (Dusa *et al*. 2006) or the kinetics of fibril formation (van Rooijen *et al*. 2009). To induce oligomerization, monomeric αSyn was incubated at 37°C with agitation. Dot blots, with the oligomer specific antibody (A11), were taken periodically to assay the formation of oligomers (Fig. 1 *A*). Oligomers were detectable as early as 30 min of incubation but consistently showed the strongest immunoreactivity after 2–3 h, during which αSyn had formed a mixture of early stage aggregates including protofibrils (Fig. 1 *Ba*). With longer incubations, immunoreactivity to the A11 antibody was lost and late stage aggregates, especially insoluble fibrils, were the major forms of αSyn present (Fig. 1 *Bb*). The A11‐positive samples, after 2–3 h of incubation, were ultracentrifuged and the supernatant loaded onto size‐exclusion chromatography to separate oligomers from monomers. The resulting purified soluble oligomers were termed *mOligomers* because they were derived directly from monomers. In addition, insoluble fibrils (produced from overnight incubation) were fragmented by a short burst of probe sonication, which subsequently recovered a separate population of soluble oligomers, termed *fOligomers*. Both *mOligomers* and *fOligomers* were positive for A11 immunoreactivity and exhibited a distinct ring‐like structure under TEM (Fig. 1 *Bc* and *Bd*). Both populations had similar size diameter (mean ± SD: *mOligomer* 14 ± 3 nm; *fOligomer* 14 ± 3 nm) and central cavity (*mOligomer* 4 ± 1 nm; *fOligomer* 4 ± 1 nm).

AUC of αSyn monomer (Fig. 1 *Ca*), *mOligomers* and *fOligomers* (Fig. 1 *Cb*) produced peak sedimentation coefficients of 1.2 S, 11.6 S and 11.3 S, respectively. Mass transformation of these peaks gave a predicted molecular mass of 8 kDa for monomer and between 250 and 260 kDa for both oligomer species, suggesting that the oligomers are composed of ∼30 monomers. The peak width at half‐height was 0.3 S for monomer, 5.4 S for *mOligomer* and 5.0 S for *fOligomer*. It is worth noting that the native αSyn tetramer (Bartels *et al*. 2011) would be expected to give a peak at ∼5 S, which is observed in the *mOligomer* sample but not the *fOligomer*, presumably because the sonication split the tetramers apart.

Fluorescence spectroscopy was used to measure the emission spectrum of Trp39 during αSyn aggregation (Fig. 1 *D*). The emission wavelength of Trp fluorescence is dependent on its exposure to the environment. Trp residues that are buried within proteins display a blue shift compared to solvent‐exposed Trp residues. Being structurally unfolded, monomeric αSyn has a freely exposed Trp39 with a fluorescence peak of 350. Upon oligomerization, the external tryptophan becomes buried resulting in a blue shift of the emission peak to 335. Interestingly, the blue‐shifted peaks for purified fibrils and *fOligomers* were 339 and 341 nm, respectively. They are still notably shifted from the monomer but significantly less than that of the *mOligomer* (Fig. 1 *D*, inset) suggesting that fibril fragmentation might recover oligomers with an altered structure.

The secondary structure of the αSyn species was measured using CD spectroscopy (Fig. 1 *Ea*) and analysed using DichroWeb (Fig. 1 *Eb*). Monomeric αSyn showed a typically unfolded protein structure, with a prominent negative band at <200 nm, whereas fibrillar αSyn had a 218 nm minimum characteristic of β sheet. The spectrum for *mOligomer* differs from fibrils because the minima is shifted to 212 and the 190 peak is more strongly positive. Similar to *mOligomers*, the spectrum for *fOligomer* clearly diverges from fibrils in wavelength intensity, particularly between 190–210 nm. However, the consistent minimum at 218 suggests the presence of β sheet rather than α helical structure. Importantly, neither *mOligomer*, nor *fOligomer* spectra could be reconstructed from linear combinations of the monomer and fibril spectra (differences were especially prominent between 200 and 210 nm), indicating that both oligomers exist as their own distinct species. Overall, these data suggests that, although the two oligomer species have similar morphologies (annular structure with approximately the same number of subunits), they display secondary structure characteristics that are different from one another; *mOligomers* are predominantly α helical, whereas *fOligomers* have more β sheet.

### αSyn oligomers and monomers were introduced into pyramidal cells

**Figure 2:**
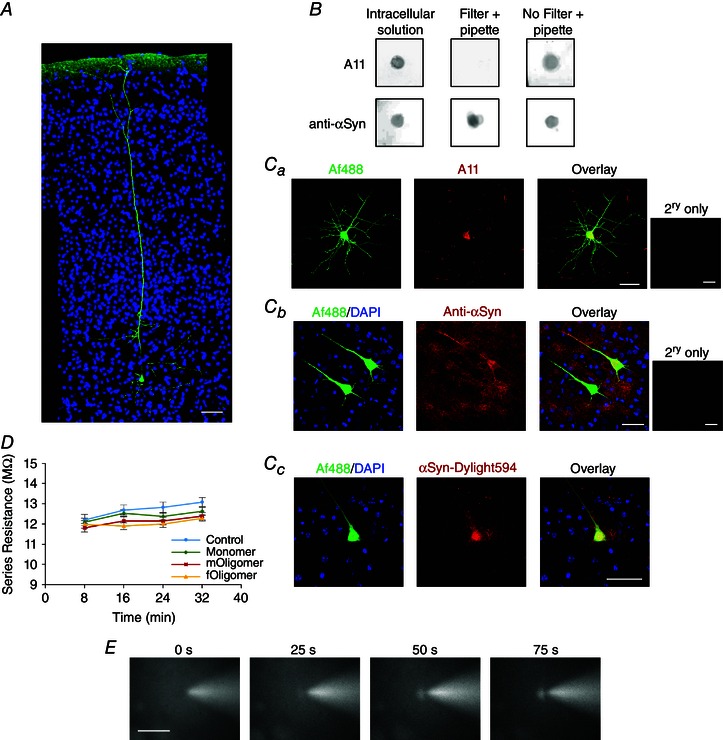
**The intracellular injection of αSyn monomer and oligomers into thick‐tufted layer 5 (TTL5) pyramial neurons** *A*, TTL5 neurons were identified by their distinct morphology and depth in the neocortex (green, Af488 dye, which is added to enable visualization; blue is the DAPI nuclear stain). *B*, dot blots showing that oligomeric αSyn stays A11‐positive when dissolved in intracellular solution and when subsequently passed out of the tip of a patch pipette. However, oligomer‐containing intracellular did not remain A11‐positive after being filtered indicating that the oligomers did not pass through the filter. *Ca*, during whole‐cell patch clamp recordings, oligomeric αSyn diffused into the recorded neuron and was detected by immunofluorescence staining (red is A11, yellow in overlay). *Cb*, paired whole‐cell recordings from TTL5 neurons, with αSyn monomer injected in the top neuron and vehicle (PBS) injected in the bottom neuron. The top neuron is stained with anti‐αSyn antibody (red, yellow in overlay), whereas the bottom neuron is unstained (green in overlay), reflecting the low level of endogenous αSyn in the soma of TTL5 neurons. *Cc*, injecting conjugated αSyn–Dylight594 demonstrated αSyn diffusion into the recorded neuron. *D*, series resistance (measured from the bridge balance, mean ± SEM) against time for cells injected with vehicle (control), monomer and both types of oligomer. Series resistance did not significantly change during recordings and did not differ depending on treatment. *E*, time lapse pictures illustrating the rapid diffusion of conjugated αSyn–Dylight594 out of the patch pipette into the recorded cell. Scale bars = 50 μm.

To investigate the effects of αSyn species on neuronal electrophysiology, oligomeric and monomeric αSyn were introduced into TTL5 pyramidal cells via the patch pipette during whole‐cell patch clamp recording. TTL5 pyramidal cells (Fig. 2 *A*) were chosen because their electrophysiological properties are well characterized (Markram, 1997 *)*, the dynamic *I*–*V* has previously been used to extract their electrophysiological parameters (Badel *et al*. 2008; Harrison *et al*. 2015) and they are affected in late stage PD (Braak *et al*. 2003; Poewe, 2008). In initial experiments, we confirmed that oligomeric αSyn preparations remained immunoreactive to the oligomer‐specific antibody (A11) once they had been dissolved in intracellular solution and passaged out of the tip of patch pipettes (Fig. 2 *B*). However, filtering (filter pore size 0.45 μm) of the intracellular solution, aiming to prevent any clogging of the recording pipette, removed A11 immunoreactivity. Thus, the intracellular solution was only filtered before the addition of αSyn oligomers. We also confirmed that both oligomers and monomers could be detected inside TTL5 pyramidal cells following whole‐cell patch clamp recording. This was achieved using either the A11 antibody (Fig. 2 *Ca*) or an αSyn protein specific antibody because little endogenous αSyn is expressed in the cell bodies of TTL5 pyramidal cells (Fig. 2 *Cb*). As an alternative method, Dylight594‐conjugated αSyn monomer was also injected into neurons to further demonstrate that the protein is able to diffuse out of the patch pipette into the cell (Fig. 2 *Cc*). This allowed us to observe the monomer moving into cells in real time with diffusion of fluorescence into the soma and apical dendrite observed within a few minutes of whole‐cell breakthrough (Fig. 2 *E*).

Including αSyn in the patch pipette could potentially lead to an increase in the series resistance during recording, compromising recording quality, particularly if the tip of the electrode becomes blocked by αSyn aggregates. Thus, we carefully monitored series resistance during recordings and rejected any recordings in which the series resistance exceeded 15.0 MΩ (Fig. 2 *D*). In the data set (18 control vehicle recordings, 20 with monomer, 19 with *mOligomer* and 13 with *fOligomer*), there was no significant difference in the series resistance at time zero (mean ± SEM breakthrough into the whole cell: control 12.2 ± 0.28 MΩ, monomer 12.1 ± 0.18 MΩ, *mOligomer* 11.81 ± 0.20 MΩ, *fOligomer* 12.0 ± 0.18 MΩ) and after 32 min of recording (control 13.1 ± 0.23 MΩ, monomer 12.6 ± 0.19 MΩ, *mOligomer* 12.38 ± 0.19 MΩ, *fOligomer* 12.3 ± 0.14 MΩ).

**Figure 3:**
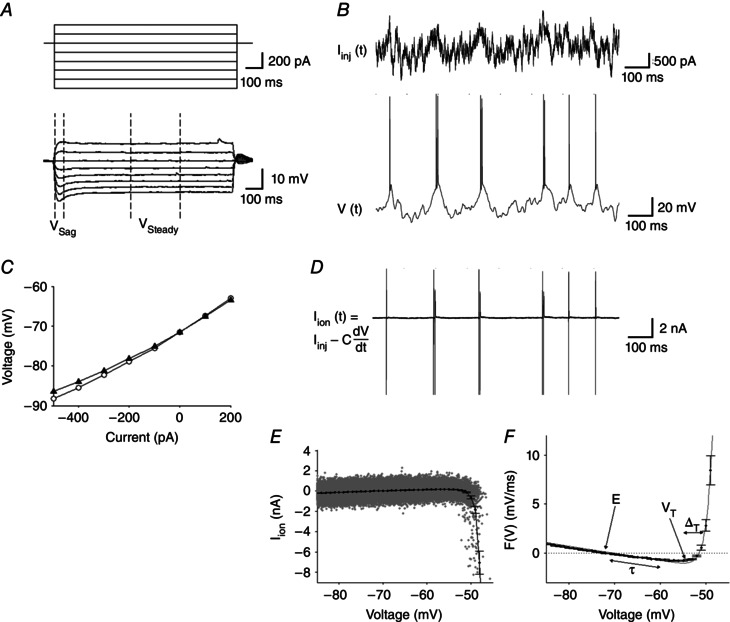
**Stimulation of neurons for electrophysiological parameter extraction** *A*, neurons were stimulated with step currents and *B*, naturalistic currents (*I*_inj_) and the subsequent voltage responses recorded. *C*, a standard *I*–*V* curve was produced from either the maximal voltage response (*V*_sag_, open circles) or during the steady‐state after *I*_H_ activation (*V*_Steady_, filled triangles). *D*, the ionic transmembrane current *I*_ion_ (*t*) was calculated from the difference between stimulated current and capacitance current. *E*, a scatter plot of *I*_ion_ *vs*. voltage (grey), with data up to 200 ms after each spike excluded. The average *I*_ion_ in 1 mV bins constitutes the dynamic *I*–*V* curve; *I*_dyn_ (black). *F*, the experimentally derived data (scatter points are the inverse of *I*_dyn_ over capacitance) can be closely fitted to a computational neuron model (line is EIF model fit) from which electrophysiological parameters can be extracted. The roles of these various parameters in determining the shape of the *I*–*V* curve are illustrated (*E*, resting membrane potential; τ, time constant; *V*_T_, spike initiation threshold; Δ_T_, spike onset sharpness).

To generate a time course for changes in electrophysiological parameters, neurons were periodically stimulated with both standard step currents (Fig. 3 *A*) and noisy currents (Fig. 3 *B*) injected in series at 8 min (start‐to‐start) intervals, over a total period of 32 min, and the subsequent voltage responses were recorded. The standard *I*–*V* curve was generated from the subthreshold response to step‐current stimuli either before (*V*_sag_) or after (*V*_steady_) the effects of *I*_H_ (Fig. 3 *C*). The dynamic *I*–*V* curve (*I*_dyn_) is equivalent to the average ionic current (Fig. 3 *D*), calculated from the difference between injected current (*I*_inj_) and capacitive current (*C*d*V*/d*t*), at a given voltage during naturalistic activity (Fig. 3 *E*; see also Methods) (Badel *et al*. 2008). The forcing function for an exponential EIF model equates to the inverse of *I*_dyn_ over capacitance, as shown by eqn (3), thus providing a rapid and accurate protocol for extracting neuronal parameters (Fig. 3 *F*).

### Parameter fittings generate reduced neuron models that accurately fit experimental voltage traces

**Figure 4:**
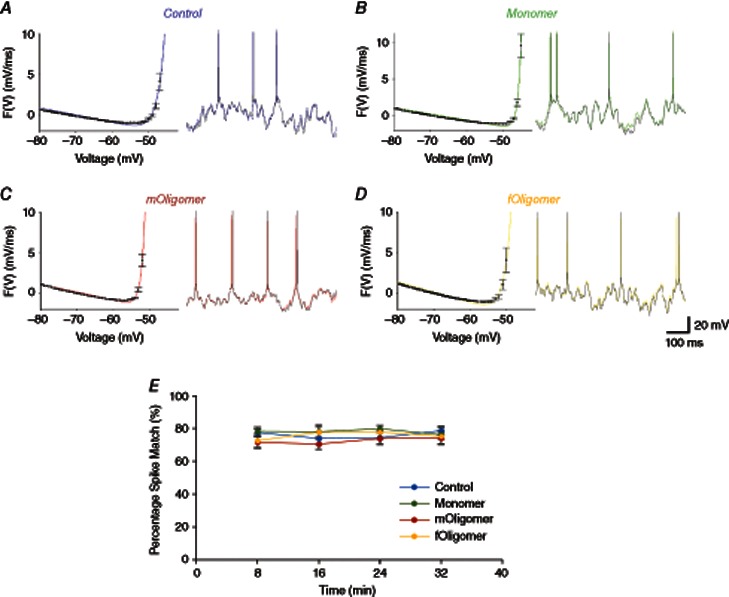
**The dynamic *I*–*V* (DIV) method allows accurate electrophysiological parameter extraction** For vehicle (*A*, control blue), monomer (*B*, green), *mOligomer* (*C*, red) and *fOligomer* (*D*, yellow) injected neurons, the EIF model (line) reliably fits the *F*(*V*) derived from the DIV curve (points). A comparison of the EIF model simulation (black line) with the experimentally recorded voltage trace (coloured line) is also shown, illustrating accurate spike prediction and very similar below‐threshold changes in membrane potential. By fitting the model to the experimental data in this way, the reliability of the extracted parameters was confirmed. *E*, mean percentage spike matches between the experimentally recorded data and the EIF simulated voltage traces. The spike matching remains >70% for all αSyn species; thus, the DIV method can be used to accurately measure electrophysiological changes in the presence of αSyn species.

To assess the quality of parameters extracted from the dynamic *I*–*V* method, the experimentally recorded voltage responses were compared with simulations using the EIF model with the extracted parameters (Harrison *et al*. 2015). The percentage of matching spikes between recorded and simulated traces (±5 ms) was >70% for control, monomer and either of the oligomeric species and did not significantly differ for each of the injected species (after 32 min, the percentage of correctly identified spikes was: control 79.1 ± 2.9 %, monomer 77.0 ± 3.6 %, *mOligomer* 72.4 ± 3.7 % and *fOligomer* 75.7 ± 5.5 %) (Fig. 4). This degree of spike matching is similar to previously reported values for TTL5 pyramidal cells (Badel *et al*. 2008; Harrison *et al*. 2015). Thus, the accuracy of parameter extraction is not compromised by dialysis of neurons with either monomeric or oligomeric αSyn. Note that the spike matching will never be 100 % as a result of spontaneous synaptic activity in experimental recordings that is absent in the simulations.

### αSyn oligomer infusion changes neuronal properties

**Figure 5:**
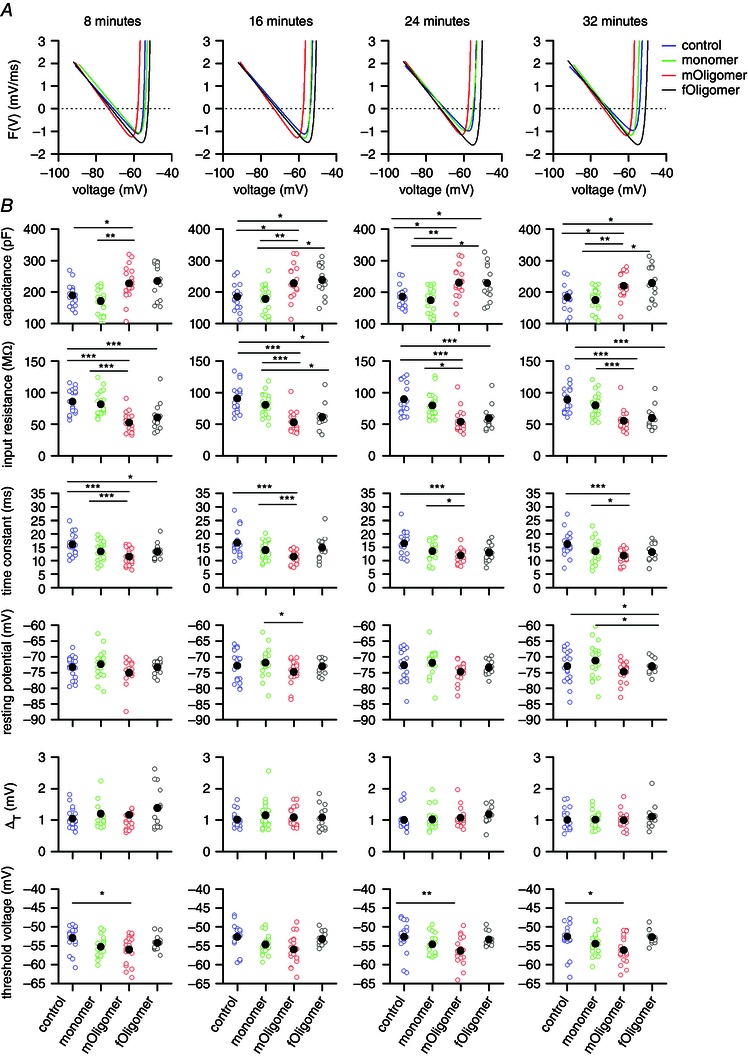
**The extracted subthreshold neuronal properties are changed by oligomer but not by monomer injection** *A*, the inverted dynamic *I*–*V* curves plotted for each αSyn species (and vehicle, control) at each time point (8, 16, 24 and 32 min). Injection of monomer has little effect, whereas both oligomers shift and change the shape of the *I*–*V* curve. *B*, quantification of subthreshold neuronal parameters: capacitance, input resistance, time constant, resting potential, ΔT and threshold voltage. Scatterplots are constructed for the time points (8, 16, 24 and 32 min, as for the dynamic *I*–*V* curves above) with open circles the data from single recordings and filled circles showing the mean values. *P < 0.05, **P < 0.01, ***P < 0.005.

αSyn monomers and oligomers were infused into TTL5 pyramidal cells via the patch pipette (all at 0.5 μm). In control recordings, an equivalent volume of the vehicle in which the monomer and oligomers were dissolved (PBS 5% v/v) was added to the intracellular solution. Dynamic *I*–*V* curves were generated for each treatment and at each time point (Fig. 5 *A*). A number of neuronal parameters were extracted from the subthreshold and pre‐spike data: membrane capacitance (*C*), membrane time constant (τ), input resistance (*R*_in_ = τ/*C*), resting membrane potential (*E*), spike threshold (*V*_T_) and spike onset sharpness (Δ_T_) (Fig. 5 *B*). The mean action potential amplitude (*A*_amp_), duration (*A*_dur_) and maximal rate of rise (*A*_rise_) were taken directly from the voltage response to noisy currents and calculated as described in Sekerli *et al*. (2004). Additional firing parameters, comprising potential between rest and threshold (*V*_T _– *E*) and spike‐initiation current *I*_spike_ = (*V*_T _– *E*)/*R*_in_, were extracted to give a measure of neuronal excitability.

In neurons infused with vehicle (control, *n* = 18), the subthreshold parameters remained stable for the duration of experiments, values were not significantly different when measured at 8 min compared to those measured at 32 min following whole‐cell breakthrough (*P* > 0.05 for all parameters) (Table 1). These parameter values are similar to those reported previously for mouse TTL5 neurons (Dani & Nelson, 2009; Oswald *et al*. 2013). Because the parameters in control neurons were stable for at least 32 min, we first investigated whether parameters significantly changed at this time point for neurons infused with αSyn species, thus giving time for the injected species to diffuse out of the patch pipette and have an effect. Injection of αSyn monomer (*n* = 20) produced no significant change (*P* > 0.05) in any of the parameters compared to control (Fig. 5 *B* and Table 1). However, there were significant changes in some of the parameters for both *mOligomer* (*n* = 19) and *fOligomer* (*n* = 13) filled neurons. There was a significant (*P* < 0.005) decrease in the input resistance (*R*_in_) of recorded cells: from (mean ± SD) 89.0 ± 22.3 MΩ in control cells to 55.1 ± 16.0 MΩ and 59.7 ± 18.6 MΩ in cells infused with *mOligomers* and *fOligomers*, respectively, which is an average reduction of ∼40 % (Fig. 5 *B* and Table 1). The membrane time constant was also significantly reduced (*P* < 0.005) from 16.1 ± 4.6 ms (control) to 11.8 ± 2.9 ms (*mOligomer*) and 13.1 ± 3.3 ms (*fOligomer*), which is an average reduction of ∼35 %. The membrane capacitance was significantly increased by an average of ∼20 % in oligomer infused cells: from 183 ± 39.8 pF (control) to 219 ± 40.3 pF (*mOligomer*, *P* < 0.005) and to 228 ± 56.1 pF (*fOligomer*, *P* < 0.05).

**Table 1:** Extracted parameter values for neurons filled with αSyn monomer, *fOligomers* and *mOligomers* and vehicle (control)

	Control				Monomer				*mOligomer*				*fOligomer*			
	8 min		32 min		8 min		32 min		8 min		32 min		8 min		32 min	
Parameter	Mean	± SD	Mean	± SD	Mean	± SD	Mean	± SD	Mean	± SD	Mean	± SD	Mean	± SD	Mean	± SD
C (pF)	189	± 35.2	183	± 39.8	171	± 43.8	172	± 40.6	227	± 53.3	219	± 40.3	247	± 58.4	228	± 56.1
*R*_in_ (MΩ)	85.7	± 19.5	89.0	± 22.3	81.4	± 18.6	79.8	± 19.7	52.3	± 15.2	55.1	± 16.0	60.5	± 22.9	59.7	± 18.6
τ (ms)	16.0	± 3.99	16.1	± 4.62	13.4	± 3.58	13.5	± 4.50	11.5	± 3.10	11.8	± 2.87	13.3	± 3.08	13.1	± 3.26
*E* (mV)	−73.4	± 3.60	−73.0	± 5.35	−72.4	± 4.33	−71.2	± 5.47	−75.1	± 4.26	−74.8	± 3.23	−73.3	± 2.20	−73.1	± 2.29
*V*_T_ (mV)	−52.9	± 3.25	−52.6	± 5.44	−55.3	± 2.59	−54.5	± 3.15	−56.0	± 3.68	−56.0	± 3.56	−54.3	± 1.98	−52.7	v1.80
*V*_T _– *E* (mV)	20.4	± 4.99	20.4	± 4.98	17.1	± 5.22	16.7	± 5.16	19.1	± 4.04	18.6	± 3.10	19.1	± 2.97	20.4	± 2.11
*I*_spike_ (pA)	249	± 78.6	243	± 87.8	220	± 76.0	225	± 89.9	385	± 107.2	358	± 95.1	343	± 98.3	367	± 104.7
Δ_T_ (mV)	1.04	± 0.33	1.00	± 0.35	1.20	± 0.42	1.02	± 0.27	1.17	± 0.92	1.00	± 0.28	1.38	± 0.69	1.11	± 0.38
*A*_amp_ (mV)	73.7	± 8.29	65.7	± 10.81	72.8	± 4.78	69.5	± 6.42	74.6	± 8.74	72.8	± 7.85	75.8	± 2.22	73.8	± 4.71
*A*_dur_ (ms)	1.30	± 0.30	1.29	± 0.29	1.32	± 0.39	1.24	± 0.32	1.00	± 0.12	1.00	± 0.12	0.95	± 0.08	0.97	± 0.10
*A*_rise_ (mV ms^−1^)	26.9	± 5.62	24.0	± 6.58	25.1	± 4.31	22.2	± 5.35	23.9	± 7.22	22.4	± 6.17	26.9	± 2.69	25.4	± 4.06

Values are the the mean ± SD at 8 and 32 min after breakthrough to whole‐cell patch clamp recording. *P < 0.05 and **P < 0.005 *vs*. control parameters at corresponding time points.

To investigate the time course for these changes in input resistance, membrane time constant and capacitance, we examined the parameters at earlier time points (8, 16 and 24 min). For each time point, the parameters for oligomer infused neurons remained significantly different from those from control neurons (Fig. 5 *B* and Table 1). This suggests that the effects of the oligomers on these parameters occurred rapidly, within the first few minutes, and then stabilized for the duration of the recordings. Interestingly, there was no significant difference between the effects of *mOligomers* and *fOligomers* on input resistance, membrane time constant or capacitance at the different time points (*P* > 0.05).

The resting membrane potential and spike onset sharpness were unaffected by αSyn species at any of the time points (Fig. 5 *B* and Table 1). The spike‐threshold voltage, however, was significantly reduced in *mOligomer* filled neurons (mean ± SD, 32 min, −56.0 ± 3.6 mV) compared to control (−52.6 ± 5.4 mV, *P* = 0.003) but not in *fOligomer* filled neurons (−52.7 ± 1.8 mV, *P* = 0.841).

The effects of *mOligomers* and *fOligomers* on subthreshold parameters are illustrated by their effects on the average dynamic *I*–*V* curves (Fig. 5 *A*). The change in membrane time constant, for example, is seen as a steeper gradient for the linear portion of the dynamic *I*–*V* curve. Similarly, the change in the threshold *V*_T_ is conveyed by the leftward shift of the *mOligomer* curve that is not seen for the *fOligomer* curve (Fig. 5 *A*). Although no significant change in resting membrane potential was found for either oligomer, their curves intersect with the control curve at around −80 mV, suggesting that αSyn oligomer‐induced conductance has a reversal potential close to this voltage.

### The reduction in input resistance is confirmed by changes in response to step currents

**Figure 6:**
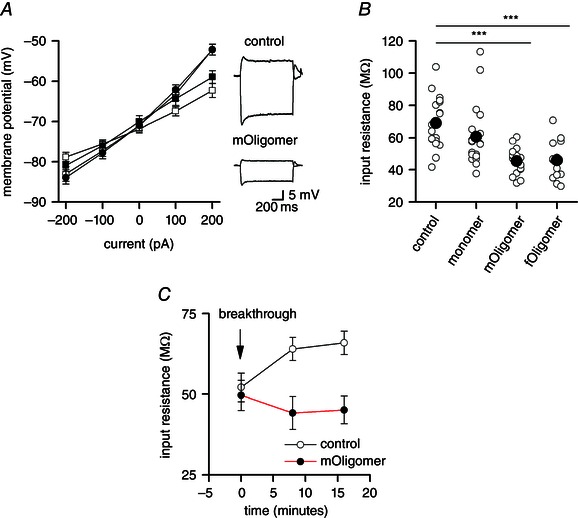
**The αSyn oligomer‐induced fall in input resistance is also observed in standard *I*–*V* curves constructed from step currents** *A*, standard step *I*–*V* curves generated from the maximum voltage response before the sag (open) and during the steady‐state (filled) after 32 min of recording. The shallower gradient at ∼0 mV for the *mOligomer I*–*V* curve (squares) compared to control (circles) illustrates a reduction in input resistance. Inset: voltage responses of neurons filled with either vehicle (control) or *mOligomer* (step currents were –400 and 100 pA). *B*, summary of input resistance measured at 32 min for control, monomer, *mOligomer* and *fOligomer* (the filled circles are the mean values). *C*, step currents were used to measure the input resistance immediately after whole‐cell breakthrough. At this early time point, the input resistance was the same for control and *mOligomer* but became significantly different over time (input resistance was measured at steady‐state). *P < 0.05, **P < 0.01, ***P < 0.005.

To corroborate the findings from the dynamic *I*–*V* method, the input resistance was also measured from the gradient, around rest, of the standard *I*–*V* curve either before or after the effects of the sag as a result of *I*_H_ activation (Fig. 6 *A*). After 32 min, the input resistance was (mean ± SEM) 69.1 ± 3.8 MΩ in the control compared to 45.4 ± 1.8 MΩ in *mOligomer* and 45.8 ± 3.4 MΩ in *fOligomer*. Thus, a reduction in input resistance similar to that measured by the dynamic *I*–*V* was observed (∼35% reduction; *P* < 0.005) (Fig. 6 *B*).

It would be predicted that the input resistance of neurons infused with either vehicle or oligomer should be the same just after breakthrough into the whole cell because there has not been sufficient time for the oligomer to diffuse into the cell. Because the standard *I*–*V* curve can be generated from a much shorter stimulus than the dynamic *I*–*V*, this method was used to measure the input resistance at time zero. As shown in Fig. 6 *D*, the input resistance was not significantly different between cells injected with vehicle (control, 52.1 ± 6.3 MΩ, *n* = 6) and *mOligomer* (49.6 ± 5.4 MΩ, *n* = 6) at time zero (*P* = 0.78) but became significantly different after 16 min (*V*_Steady_: control *R*_in_ 58.7 ± 3.2 MΩ *vs*. *mOligomer R*_in_ 44.2 ± 2.6 MΩ, *P* = 0.001) (Fig. 6 *C*), confirming that the changes in input resistance occur within a short period after breaking through into the whole cell.

In a subset of cells, the concentration of *mOligomer* in the intracellular solution was increased from 0.5 to 1.5 μm (*n* = 4) to determine whether the effects on parameters were concentration‐dependent. A fall in input resistance was still observed for *mOligomer* at this higher concentration (not shown); however, the change was not significantly greater than that observed with a lower concentration. Increasing the monomer concentration from 0.5 to 1.5 μm (*n* = 7) still did not lead to a significant change in input resistance.

### Oligomeric αSyn reduces neuronal excitability

**Figure 7:**
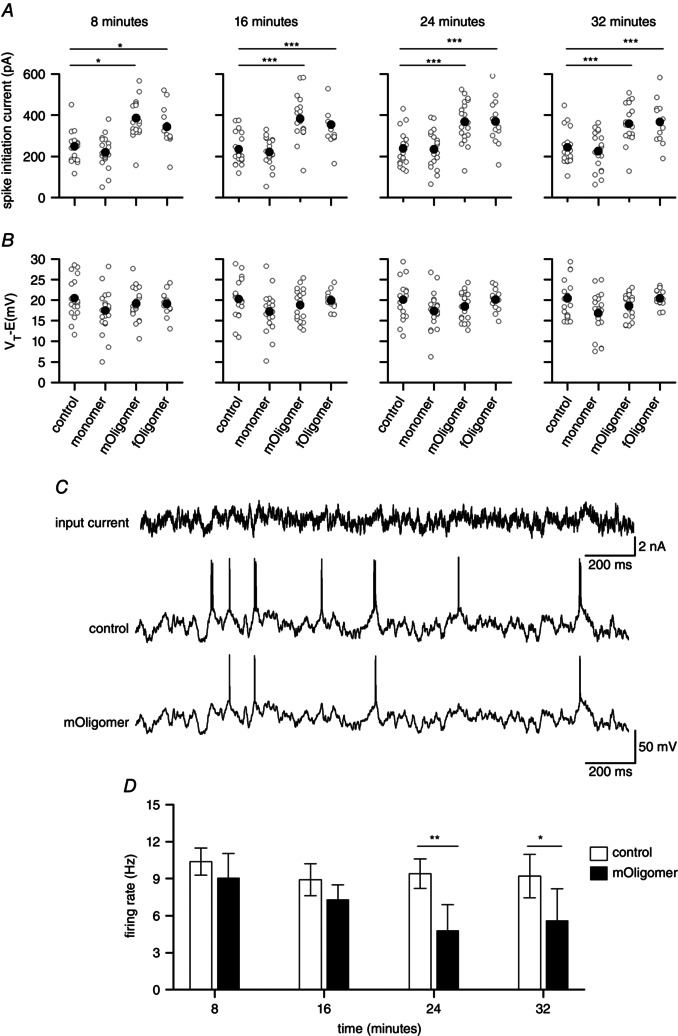
**Neurons infused with αSyn oligomers show a marked reduction in excitability** *A*, scatterplots of spike initiation current (*I*_Spike_), the amount of current required for a resting neuron to reach threshold, at different time points from whole‐cell breakthrough for control; monomer and oligomer‐infused cells. *B*, scatterplots of the voltage difference between resting and threshold (*V*_T_ – *E*) from whole‐cell breakthrough for control; monomer and oligomer‐infused cells. *A* and *B*, open circles are values from individual recordings and the filled circles are mean values. *C*, example voltage responses to a noisy current stimulus (gain = 400 pA) for a cell filled with vehicle (PBS, control) and a cell filled with *mOligomer*. *D*, bar chart plotting the mean firing rate for neurons infused with *mOligomer* or vehicle against the time from whole‐cell breakthrough. *P < 0.05, **P < 0.01, ***P < 0.005.

With a reduction in input resistance, the voltage response to a given current will decrease. Thus, it would be expected that a larger current (*I*_spike_) would be required to reach the action potential firing threshold. For neurons infused with either oligomeric species, there was a more than ∼60 % increase in *I*_spike_ amplitude compared to control (*P* < 0.005) (Fig. 7 *A*). However, the potential difference between the resting voltage and threshold (*V*_T _– *E*) was not significantly affected by αSyn treatments (Fig. 7 *B*).

**Figure 8:**
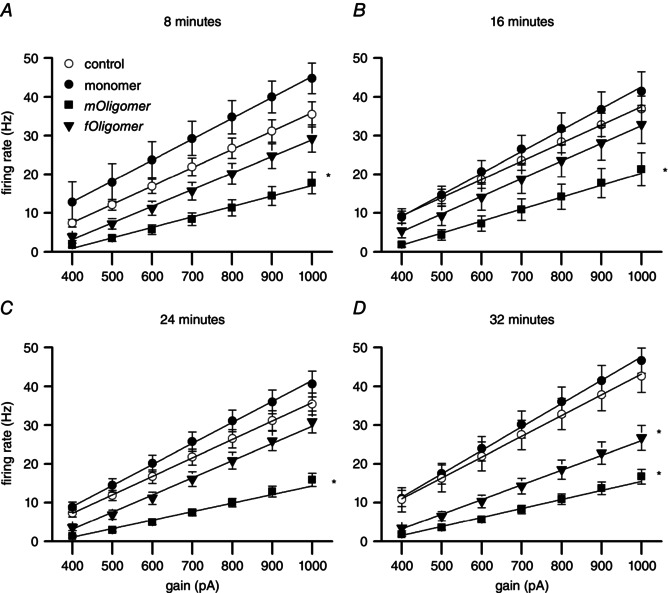
**EIF models predict the reduction in firing rate produced by αSyn oligomers** *A*–*D*, firing rate plotted against input current gain factor from EIF models informed by parameters from control, monomer, *mOligomer* and *fOligomer* filled neurons at different time points. The linear regressions at each time point were subject to analysis of covariance; those marked with an asterisk (*) had a significantly different intercept and slope than either control or monomer. This indicates that, for the *mOligomer* at all‐time points (*P* < 0.005) and *fOligomer* at 32 min (*P* < 0.05), both the overall firing rate (intercept) and the change in firing rate with increasing gain (slope) were significantly different from control and monomer.

It would be predicted that the firing rate of oligomer infused cells would be lower than cells injected with vehicle, for a given current input. In most recordings, this could not be directly observed because the gain factor for the injected noisy currents was altered to maintain the same firing rate (i.e. a consistent firing rate is required for accurate parameter measurement; see Methods) (Badel *et al*. 2008). It was noted, however, that a higher gain factor was often required for oligomer filled cells than for cells injected with vehicle to produce the same firing rate. In a subset of recordings, the gain of the current input was set at the same level (400 pA) for both control cells and interleaved oligomer infused cells to allow the firing rate to be directly compared (Fig. 7 *C* and *D*). After 32 min, the mean ± SEM firing rate was 9.4 ± 0.54 Hz (*n* = 5) in control neurons compared to only 5.6 ± 1.14 Hz in *mOligomer* filled neurons (*n* = 5), which is a marked (*P* < 0.005) decrease in firing rate (on average, a decrease of ∼40%) (Fig. 7 *D*). To investigate this further, simplified neuron models fitted to the experimental parameters were simulated with increasing input current gain factors (from 400 to 1000 pA in steps of 100) (Fig. 8). This linear increase in firing rate was significantly reduced (as determined by analysis of covariance) in simulations using parameters from either *fOligomer* (*P* < 0.05) or *mOligomer* (*P* < 0.005) filled neurons compared to either control or monomer (Fig. 8). Interestingly, the simulated firing rate for *mOligomer* was reduced after 8 min, whereas *fOligomer* only had a significant effect after 32 min. This later reduction was also significantly weaker than produced by *mOligomers* (the gradients of the linear regressions were significantly different from one another; *P* < 0.005).

### Oligomeric αSyn reduces action potential duration

**Figure 9:**
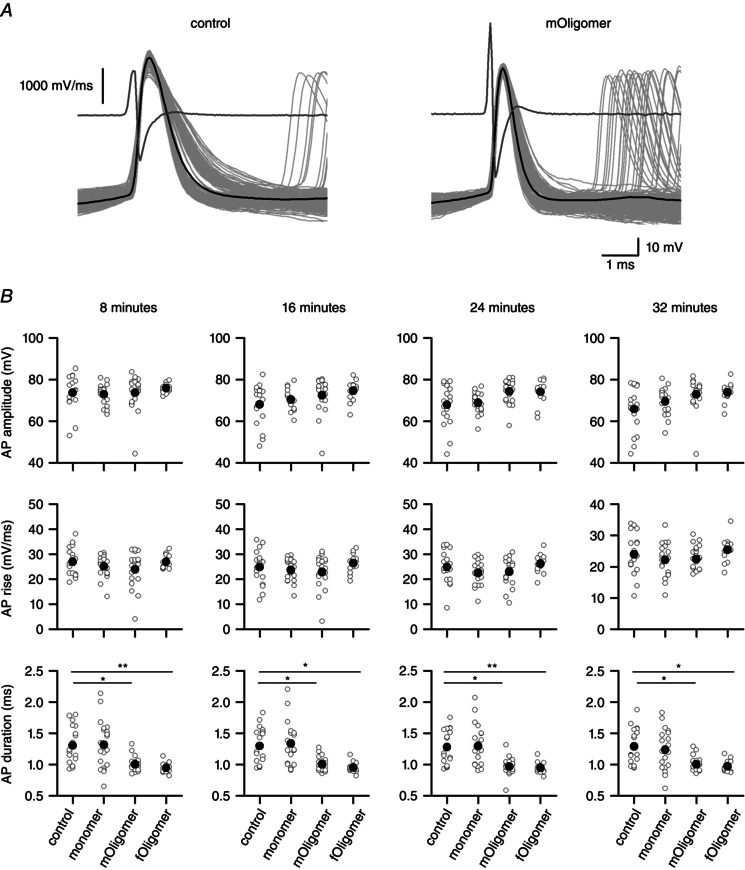
**Action potential duration is reduced in neurons infused with αSyn oligomers** *A*, the average spike (black) was generated from isolated spikes in voltage responses to naturalistic current with an interspike‐interval greater than 200 ms from a vehicle filled cell (control) and from a cell infused with *mOligomer*. The individual spikes are in grey. The spike initiation threshold was calculated using the peak second‐derivative method (Sekerli *et al*. 2004), shown in dark grey. *B*, scatterplots of action potential (AP) amplitude, maximum rate of action potential rise and action potential duration for each time point (8, 16, 24 and 32 min) for all recordings in the data set. The open circles are values from individual recordings and the filled circles are the mean values. The duration of the action potential was significantly shorter for oligomer‐filled neurons than for either vehicle or monomer‐filled neurons. *P < 0.05, **P < 0.01, ***P < 0.005.

We investigated whether αSyn oligomers had any effect on the action potential by measuring a number of parameters that define the action potential waveform (Fig. 9 *A*). There was no significant difference in action potential amplitude. However, action potential duration (*A*_dur_) was significantly reduced by both *mOligomer* (8 min; *P* < 0.005) and *fOligomer* (*P* < 0.005) but not by monomer (*P* = 0.606) (Fig. 9 *B*). This decrease in duration was not associated with an increase in the maximal rate of rise (*A*_rise_) of the action potential but, instead, was associated with a faster rate of repolarization.

## Discussion

The present study aimed to combine the structural characterization of αSyn oligomeric species with detailed insight into their time‐dependent effects on electrophysiological parameters measured in neocortical neurons. We generated two distinct species of oligomer with similar morphologies but different secondary structures. We then took the unique approach of introducing these oligomeric species directly into neurons during whole‐cell patch clamp recording. This approach has major advantages: it only requires a small amount of oligomeric species compared to extracellular incubation and it allows changes in neuronal parameters to be measured in real time. Because the recorded neurons are healthy before recording and the introduction of oligomers, changes in neuronal properties can be observed in real time unlike with extracellular pre‐incubation. In the majority of experiments, we used a concentration of 0.5 μm in the pipette solution for both αSyn monomers and oligomers. We based this on previous studies showing extracellular effects of αSyn oligomers within a concentration range of 0.1–0.5 μm (Diogenes *et al*. 2012; Martin *et al*. 2011; Tosatto *et al*. 2012). The precise concentration of αSyn oligomers inside neurons in pathological states is unclear, although it will almost certainly be higher than in the extracellular space.

### Soluble αSyn oligomers isolated during the aggregation process or recovered from fibril fragmentation have similar morphologies but differ in secondary structure

Two populations of αSyn oligomer were isolated from different stages of the aggregation process. Oligomers that were formed directly from monomer under fibrillizing conditions, *mOligomers*, were produced in accordance with a protocol used previously to give structural and kinetic information (Lorenzen *et al*. 2014). This soluble *mOligomer* species has a ring‐like shape (annular), is ∼14 nm in diameter with a central cavity of 4 nm and is comprised of ∼30 monomer subunits, which are properties similar to those reported previously (Lashuel *et al*. 2002).

We also found that soluble, A11‐positive, oligomers could be recovered after subjecting fully formed fibrils to sonication, *fOligomers*. These oligomers had a very similar morphology to *mOligomers* but differed in their secondary structure (discussed below). Fibril fragmentation is a key determinant of the physiological impact of amyloid proteins (Tanaka *et al*. 2006; Xue *et al*. 2010; Wang *et al*. 2011 *b*) and may be a more important process than elongation. For example, amyloid proteins with a slow growth rate but increased brittleness are more cytotoxic than rapidly growing stable amyloids (Xue *et al*. 2009). It was also observed by Xue *et al*. (2009) that fibril fragmentation not only generated new seeds that rapidly increased the fibril load, but also created a variety of small fibrilliar species with pathological properties. The oligomeric population that we recovered from fibril fragmentation, *fOligomers*, could represent one of these pathological species.

The *mOligomer* CD spectrum (minimum of 212 nm) suggests that this species has a lower β sheet content compared to fibrils and is predicted by DichroWeb to be predominantly α helical in structure. The N‐terminal region of αSyn is capable of forming α helixes and this appears to be vital for lipid–membrane interactions (van Rooijen *et al*. 2009). This region also appears to be folded in *mOligomers* because the fluorescence spectrum from Trp39 is strongly blue shifted, significantly more so than for fibrils. Thus, the microenvironment in this region appears altered in the oligomeric state. The *fOligomers*, recovered from sonicating fibrils, did not produce the same level of blue shift or have the same amount of α helical content as *mOligomers*, suggesting that *fOligomers* retain elements of the fibril structure that are not present in *mOligomers*. The distinct structure of *mOligomers* points towards them being a species that is off‐pathway from fibrils; this idea that is supported by their ability to inhibit the kinetics of amyloid formation in a concentration‐dependent manner (Timothy J. Kaufmann and Teresa J. T. Pinheiro, unpublished data; Lorenzen *et al*. 2014).

### αSyn oligomers reduce input resistance and increase cell capacitance

We have taken the novel approach of dissolving αSyn species into intracellular recording solution and then injecting them directly into the soma of neurons during whole‐cell patch clamp recording. The intracellular recording solution contains an ATP regenerating system (phosphocreatine), as well as 2 mm ATP. Such a system is important because it prevents the ‘rundown’ of many voltage‐gated and ligand‐gated ion channels during whole‐cell dialysis (Chen *et al*. 1990; Rosenmund & Westbrook, 1993). However, it could occlude the effects of a fall in ATP production as a result of any disruption of mitochondrial function produced by αSyn species.

After injecting αSyn monomer and oligomeric species into pyramidal neurons, electrophysiological parameters were extracted at regular intervals using both standard and dynamic *I*–*V* methods. The dynamic *I*–*V* curve has been shown to reliably and accurately fit reduced neuron models (Badel *et al*. 2008). The reliability of this fit was confirmed by simulating voltage traces with extracted parameters and comparing them with the original recorded traces; in all experiments, the percentage of matching spikes was high and comparable to previous publications (Fig. 3) (Badel *et al*. 2008; Harrison *et al*. 2015).

Using the dynamic *I*–*V* method, we found that neurons injected with either species of oligomeric αSyn showed three major changes that occurred rapidly after whole‐cell breakthrough: a marked reduction in input resistance (*R*_in_), a fall in membrane time constant (τ) and an increase in cell capacitance (*C*). The values for *R*_in_, τ and *C* for control (vehicle filled) neurons were similar to those previously reported for TTL5 pyramidal neurons in mouse neocortical slices (Dani & Nelson, 2009; Oswald *et al*. 2013). Although we have no clear measure of the rate of diffusion of oligomer into neurons, Dylight 594 labelled monomer could be detected within the soma and dendrite within a few minutes. Oligomer is clearly much larger than the monomer and thus will diffuse more slowly. However, we know that it does diffuse into neurons as a result of the immunohistochemistry and the observed changes in neuronal properties and thus must be present within the soma within 8 min of breakthrough because this is when we take our first measurement.

It is interesting that the effects of oligomer occur rapidly, reach a maximum and then there are no further changes over the duration of the recordings. This is unexpected because, instead, a slow change in the neuronal parameters during the recordings would be predicted as the oligomer diffuses into the recorded cells and the intracellular concentration increases. One possible explanation is that, at 0.5 μm, the effects of oligomer are at a maximum. This is supported by the actions of larger concentrations of oligomer (1.5 μm) that did not have any significantly greater effect and could be tested further by using lower concentrations of oligomer to determine whether there is a slower time course.

The changes produced by oligomeric αSyn, were not observed with monomer, even if the concentration of monomer was increased to 1.5 μm. This is interesting because it suggests that, over the time period of the experiments (up to 2 h for some recordings), the introduced monomer does not oligomerize inside the cell. An alternative explanation is that any oligomer formed inside the cell by monomer is in a different cellular compartment to the oligomer when it is directly introduced and thus has different activity.

The oligomer‐mediated reduction of *R*_in_ will supress neuronal excitability because the same amount of input current will produce a smaller voltage change. Thus, a greater amount of current (*I*_Spike_) is required to reach action potential threshold and the firing rate is reduced. However, the slow onset for the fall in firing rate (particularly for the *fOligomer*) suggests that other mechanisms may also be involved (see below). The mechanism underlying the fall in *R*_in_ could be the insertion of channels into the membrane. Increasing evidence suggests that αSyn oligomers can directly form pore‐complexes that are able to permeabilize lipid membranes as shown both *in vitro* (Volles & Lansbury, 2002; Kim *et al*. 2009; Schmidt *et al*. 2012) and in cell culture (Feng *et al*. 2010; Tosatto *et al*. 2012; Mironov, 2015). It has also been reported that this pore formation can be very rapid, occurring within the first 5 min of incubation (Mironov, 2015). In our experiments, the oligomer‐mediated change in *R*_in_ was not present at time zero (whole‐cell breakthrough) but was present after 8–16 min of recording. In common with other studies (Robinson and Cameron, 2000), we found that the input resistance in control cells increased during whole‐cell recording and reached a steady‐state after ∼10–15 min, probably as a result of whole‐cell dialysis. This increase was not observed when cells were infused with oligomer, suggesting that the effects of oligomer (e.g. pore insertion) counteracted the effects of cell dialysis. Alternatively, αSyn oligomers could interact with existing voltage‐gated or ligand‐gated ion channels that are already in the membrane or modify the membrane trafficking of such channels. For example, it has been reported that there is cross‐talk between αSyn and voltage‐gated K^+^ channels (Mironov, 2015) and β amyloid oligomers can change the activity of Kv 1.3 channels, whereas monomers have no effect (Lioudyno *et al*. 2012). The recruitment of voltage‐gated K^+^ channels to the membrane or changes in K^+^ channel activation would be consistent with the observed speeding of action potential repolarization. Although oligomers produced a marked change in *R*_in_ consistent with an increase in membrane conductance, there was no significant change in the membrane potential (at 32 min, control −72.3 *vs*. −74.5 and −73.1 mV, for *mOligomers* and *fOligomers*). One possible explanation is that the reversal potential for the induced conductance change is close to the resting potential. It has previously been reported that the pores formed by αSyn are non‐selective cation channels (Feng *et al*. 2010; Schmidtt *et al*. 2012; Tosatto *et al*. 2012), which would be expected to depolarize the membrane potential. By contrast, if αSyn interacts with K^+^ channels, the reversal potential for K^+^ is around −80 mV and close to the resting potential, which was the point where the dynamic *I*–*V* curves intersected (Fig. 5 *A*), and would be expected to produce only a small hyperpolarization. An alternative explanation is that inclusion of oligomers in the recording solution changed the liquid junction potential, which occluded any change in the resting potential. Although we cannot completely rule this out, it is not probable because: (1) the amount of oligomer added to the intracellular solution was very low; (2) the change in liquid junction potential would have to be opposite and equal to the change in membrane potential to occlude it; and (3) there was no change in the difference between the resting potential (*E*) and the threshold potential (*V*_T_), which would be altered if the resting potential had been changed.

The reduction in firing rate developed more slowly than the rapid change in input resistance (particularly for *fOligomer*). This implies that that the change in firing rate is not solely a consequence of the fall in input resistance and, instead, may depend on the involvement of other slower processes; for example, changes in K^+^ currents or Na^+^ channel activation, which may be delayed if occurring in more distal compartments.

Oligomeric αSyn also produced a significant increase in cell capacitance (∼20 %). This result would not be predicted because the build‐up of oligomeric aggregates inside the recorded neuron could occlude neuronal process and thus reduce the effective capacitance of the cell as measured from the patch electrode. It has been suggested, however, that amyloid oligomers can insert into lipid bilayer layers forcing apart the head groups, leading to a fall in the thickness of the membrane and increasing capacitance (Sokolov *et al*. 2006).

### The different αSyn oligomers have similar effects on neuronal properties

Despite their structural differences, the two oligomers (*mOligomers* and *fOligomers*) produced very similar changes in the electrophysiological properties of TTL5 neurons. Because both oligomeric species have a similar morphology and are of a similar size, they may also share the same pathological mechanism. Although there were differences in their secondary structures, this appeared to only have subtle effects on their activity. For example, there appear to be some differences in their effects on the shape of the dynamic *I*–*V* curve and differences on their effects on neuronal excitability suggesting that *mOligomers* had a greater and faster effect than *fOligomers*. It has previously been reported that the membrane permeabilizing activity of oligomers is dependent on the structural flexibility of their hydrophobic core (Campioni *et al*. 2010). Differences in Trp39 fluorescence and CD spectra indicate potential differences in core rigidity between *mOligomers* and *fOligomers* that could have an impact on their ability to interact with membranes.

In conclusion, the electrophysiological impact of two structurally characterized αSyn oligomers has been investigated, in detail, for cortical neurons. These two oligomeric species shared a similar morphology and produced similar effects on the input resistance and excitability of neurons. These changes could potentially affect network activity and thus contribute to the early changes in cognition observed in PD.

## Additional information

### Competing interests

The authors declare that they have no competing interests.

### Author contributions

TJK, PMH, MJER and TJTP conceived and designed the experiments. All experiments were carried out by TJK in the laboratories of MJW and TJTP. Data were analysed by TJK with the assistance of PMH. The manuscript was written by TJK and MJW with assistance from PMH, MJER and JTTP. All authors have approved the final version of the manuscript and agree to be accountable for all aspects of the work. All persons designated as authors qualify for authorship, and all those who qualify for authorship are listed.

### Funding

We acknowledge funding received via a BBSRC studentship to TJK.
